# Adjoint-Assisted Shape Optimization of Microlenses for CMOS Image Sensors

**DOI:** 10.3390/s24237693

**Published:** 2024-11-30

**Authors:** Rishad Arfin, Jens Niegemann, Dylan McGuire, Mohamed H. Bakr

**Affiliations:** 1 Department of Electrical & Computer Engineering, McMaster University, Hamilton, ON L8S 4K1, Canada; mbakr@mcmaster.ca; 2 Ansys Canada Ltd., 1700-1095 West Pender Street, Vancouver, BC V6E 2M6, Canada; jens.niegemann@ansys.com (J.N.); dylan.mcguire@ansys.com (D.M.)

**Keywords:** adjoint sensitivity analysis (ASA), optimization, microlenses, CMOS image sensor

## Abstract

Recently, there have been significant developments in the designs of CMOS image sensors to achieve high-resolution sensing capabilities. One of the fundamental factors determining the sensor’s ability to capture high-resolution images is its efficiency in focusing the visible light onto the photosensitive region of the submicron scale. In most CMOS imaging technologies, this is typically achieved through microlenses. Light collection under diverse conditions can be significantly improved through the efficient design of microlenses. While the optimization of microlenses appears to be imperative, achieving efficient designs of microlenses for high-density pixels under various conditions remains a significant challenge. Therefore, a systematic optimization approach is required to accelerate the development of efficient microlenses with enhanced optical performance. In this paper, we present an approach to optimize the shape of CMOS microlenses through adjoint sensitivity analysis (ASA). A novel figure of merit (FOM) is developed and incorporated into the optimization process to enhance the light collection. The gradient of the FOM is computed iteratively using two field simulations only. The functionality and robustness of the optimization framework are thoroughly evaluated. Furthermore, the performance of the optimized CMOS microlenses is compared to that of the conventional microlenses. The adjoint-assisted optimization framework presented here can be further used to develop efficient optical devices that perform optical manipulation such as concentrating, bending, or dispersing light in compact imaging systems.

## 1. Introduction

The recent advancements in CMOS image sensors enable their wide utilization in different imaging systems used in digital cameras, surveillance, automobiles, and medical imaging devices [1,2]. The adoption and integration of CMOS image sensors in various imaging and sensing systems are largely attributed to their miniaturized features. While the pursuit of miniaturization continues, the size of the pixel is further reduced to achieve images of high resolution. However, the primary challenges in attaining images from high-density pixels are twofold. First, as the pixel size is reduced, the photosensitive region located directly beneath the respective pixel is also shrunk. Consequently, the intensity of light in the photosensitive region for each pixel is also reduced. This leads to a degradation in signal strength, resulting in poor image quality [3,4]. Second, in conventional CMOS image sensors, pixels corresponding to the primary colors, i.e., RGB, are equipped with microlenses to concentrate the incoming light onto the photosensitive regions [5]. However, with the reduction in pixel size, the dimensions of these microlenses become comparable to the wavelengths of the visible spectrum [6]. This causes the incoming light to diffract and spread out within the region between microlenses and photosensitive areas. As a result, the light is collected by the photodiodes of neighboring pixels. This reduced directivity of light affects color accuracy and results in reduced image quality [7]. Thus, to improve the focusing of light with enhanced intensity onto the photosensitive region, efficient design of CMOS microlenses becomes imperative.

Several optimization strategies have been adopted to improve the performance of CMOS microlenses presented in [1,5,8,9,10,11,12,13]. For instance, a detailed investigation is performed in [8] to determine the optimum design parameters and lateral position of microlenses by analyzing the crosstalk and sensitivity distribution. The study reported in [9] attempted to optimize the radius of curvature (ROC) for microlenses using an automated optimization algorithm. Different geometric profiles for CMOS microlenses are studied in [5] to enhance the collection of light in the photodiodes. A recent study in [10] determined the optimal distance between the microlens array and CMOS photodiode to mitigate crosstalk by optimizing the design parameters using a commercial optimization tool. While the studies reported in [5,8,9,10] primarily used optical ray tracing methods to optimize CMOS microlenses, electromagnetic field analysis offers higher accuracy for CMOS image sensors with reduced pixel size [11]. The studies reported in [11,12] employed the finite-difference time-domain (FDTD) to accurately evaluate the response of a CMOS image sensor under broadband excitation. Furthermore, the study presented in [13] achieved efficient microlense configurations by optimizing the ROC for different pixel sizes and adjusting the position of microlenses to accommodate variations in incidence angle. However, most of these previous studies focused on improving the optical performance of the CMOS image sensor by investigating and comparing the effects of adjusting the position and ROC of microlenses. This poses two key challenges in the efficient development of CMOS microlenses. First, finding efficient designs of microlenses for different CMOS optical stacks often relies on extensive investigations and comparative studies, making the process inefficient. Second, the approaches may result in suboptimal performance as only a small portion of the overall design space is explored. Therefore, a systematic and efficient optimization methodology is required to navigate a larger design space and achieve designs of CMOS microlenses with enhanced performance.

Motivated by the drawbacks reported in the previous studies [1,5,8,9,10,11,12,13], we employ an optimization framework that uses a derivative-based approach to systematically optimize the shape of CMOS microlenses. The approach uses the gradient information of the figure of merit (FOM) to carry out the optimization process. The gradient of the FOM is computed efficiently through the exploitation of adjoint sensitivity analysis (ASA) [14,15,16]. ASA is a powerful method that computes the gradient of the FOM with respect to all design parameters. This gradient computation is performed using only two full-field electromagnetic simulations per iteration regardless of the number of parameters. The ASA has been used widely for design sensitivity and optimization in microwave applications [14,17,18,19,20]. In recent years, adjoint-driven optimization has also been employed in inverse design processes to optimize photonic devices [21,22,23,24,25,26,27,28,29,30,31,32]. For instance, dielectric microlenses are developed in [33] using adjoint-based optimization for photodetector applications. Furthermore, a recent study optimized the shape of microlenses in [34] using an FEM-based shape gradient technique for photonic nanojet applications.

In this work, we present a systematic and efficient optimization approach of CMOS microlenses using ASA. A novel FOM is introduced and developed in the optimization framework to maximize the intensity of light within a specified region. Ansys Lumerical FDTD [35] is employed, as a numerical solver, to carry out all the electromagnetic simulations. LumOpt [21,36], an open-source package, is utilized to conduct the adjoint-driven optimization process. We have significantly extended the existing LumOpt codebase to incorporate the newly devised FOM and modified the adjoint formulation for free-space photonic problems. These substantial developments in LumOpt expand its capabilities and enable the accurate computation of gradients using adjoint fields for new classes of free-space optical devices, such as microlenses. The curvature boundary or surface geometry that defines the shape of the microlenses is systematically and gradually perturbed during the optimization process. The functionality and robustness of the optimization methodology are evaluated by varying the source condition, employing different sets of parameterizations for microlenses, and considering different focusing regions. Furthermore, we evaluated the angular response (AR) of the optimal microlenses and contrasted the performance with the conventional design of microlenses. The adjoint-assisted optimization framework presented in this paper can be further used to effectively tackle free-space photonic problems [23,24,37] and accelerate the development of new classes of efficient optical and photonic devices.

The rest of the paper is organized as follows: Section 2 discusses the computational framework, device modeling, and optimization methodology employed for the CMOS microlenses. Section 3 introduces the powerful adjoint method and describes adjoint-aided gradient computation in the context of electromagnetics. A comprehensive and detailed evaluation of the optimization methodology is presented in Section 4. Finally, to summarize the study, concluding remarks are listed in Section 5.

## 2. Optimization of CMOS Microlenses

**Figure 1:**
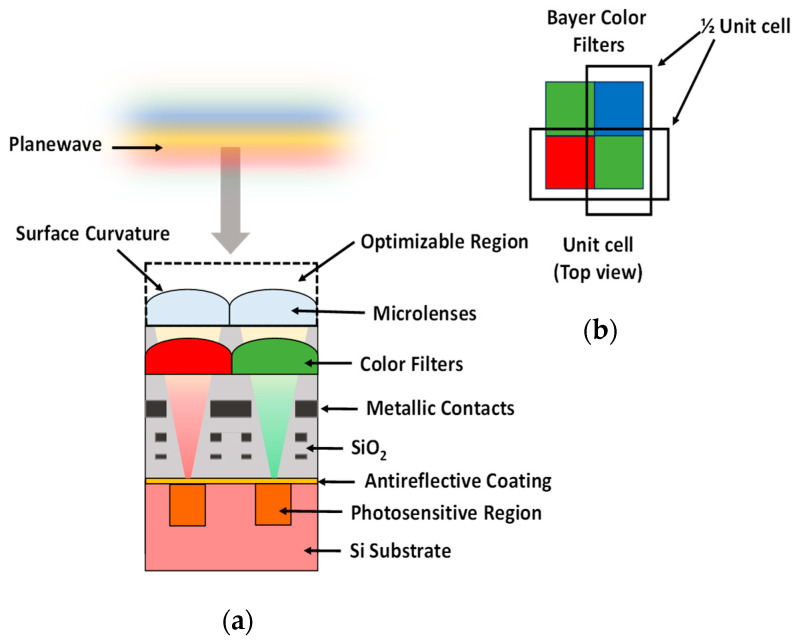
(**a**) A cross-sectional view of CMOS image sensor; (**b**) a unit cell of Bayer color filter having primary colors: red, green, and blue (RGB) in mosaic-like pattern.

Microlenses, as an integral component of the CMOS image sensor, are positioned above the photosensitive region, as shown in Figure 1. The primary function of the CMOS microlenses is to focus the incoming visible light directly onto the photodiodes of the pixels. The recent trend towards miniaturized compact devices and the demand for high-resolution images drive the demand for smaller pixels. However, this has a number of undesired consequences. For one, the collection of light is reduced, leading to a decrease in light intensity per pixel. Additionally, a shift towards smaller pixels also increases the likelihood of optical crosstalk. These shortcomings lead to an overall degradation of image quality. The enhanced focusing of light in the active region of the pixels can be ensured through efficient designs of microlenses. Hence, it is necessary to develop a methodological approach to accelerate the optimization of microlenses. In this section, first, we briefly discuss the computational methodology to demonstrate the systematic optimization of CMOS microlenses. Furthermore, we elaborate on the primary building blocks of the optimization process.

### 2.1. Computational Methodology

**Figure 2:**
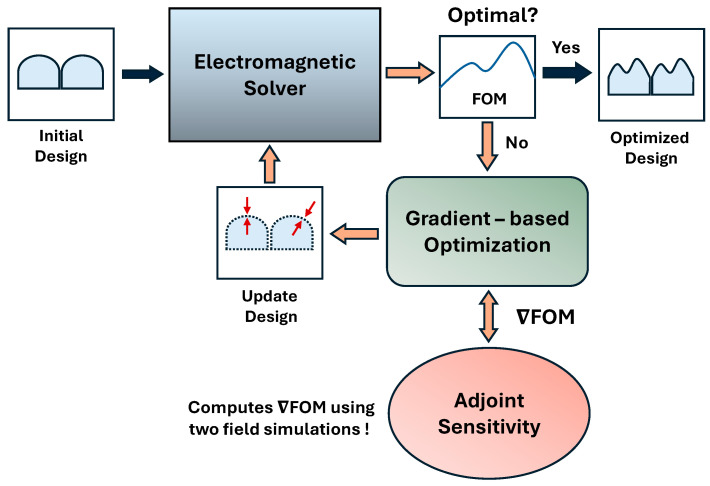
Computational methodology for systematic optimization of CMOS microlenses.

The computational methodology for systematic optimization includes two primary building blocks, as shown in Figure 2. Firstly, it has an electromagnetic solver that computes the fields and evaluates the optical performance of the CMOS image sensor. Secondly, it has an optimizer (or optimization algorithm) that maximizes the FOM with respect to the design parameters. For instance, we consider light intensity in a specified region as FOM in our study. The defined FOM effectively quantifies the overall optical performance of the CMOS image sensor. The optimizer uses FOM sensitivity (or gradient of FOM with respect to design parameters) to explore the design space and identify the optimal design parameters for microlenses that maximize the FOM. The FOM sensitivity is efficiently computed using ASA to update the design parameters of microlenses during optimization iterations.

### 2.2. Device Modeling

In this study, we use the FDTD method to solve Maxwell’s equations. This method is widely used as it is highly efficient in simulating complex photonic structures. The FDTD is well suited for our application as it considers diffraction effects due to reduced pixel size [11]. Moreover, CMOS image sensors are exposed to a broad range of wavelengths for imaging purposes. Therefore, a wideband time-domain simulation is preferred to ensure the proper evaluation of the optical performance.

We have numerically implemented a CMOS image sensor in a 2D computational grid using FDTD. This numerical model includes five different segments, as previously shown in Figure 1. A Silicon (Si) substrate is positioned at the bottom of the structure. We aim to maximize the light absorption at the surface of this layer. Above the substrate’s surface lies an anti-reflection coating, having a finite thickness of 70 nm. In the intermediate segment of the structure, integrated metallic interconnects used for wiring purposes are visible within the dielectric layer of Silicon dioxide (SiO_2_). Bayer color filters [38], having a mosaic-like pattern, are used within the dielectric SiO_2_ layer to allow the transmission of primary colors, i.e., RGB, to their respective pixels. Each of the color pixels is 2 μm wide. Finally, the optimizable microlenses are placed at the top of the CMOS structure to focus the incoming light into the photosensitive region within the Si substrate. This photosensitive region is 1 μm wide, resulting in a fill factor of 25%. A plane wave excitation is used as the illumination source. A perfectly matched layer (PML) is employed as an absorbing boundary condition at the top and bottom of the structure. Furthermore, the Bloch-periodic boundary condition is utilized to represent the repetitive unit cells of the CMOS image sensor.

### 2.3. Shape Optimization

The primary focus of our work is to optimize microlenses in a systematic and efficient way that improves the overall optical performance of the CMOS image sensor. For this purpose, we carried out the shape optimization of the CMOS microlenses. The position of the interface between the microlenses and air is iteratively adjusted to improve the FOM. While the landscape of the FOM for a photonic problem is mostly nonconvex [39], a good initial design strongly influences the optimization process to reach an optimal design. Therefore, we chose an optical stack for the CMOS image sensor that includes the conventional microlenses. The conventional CMOS microlenses used as an initial design (or starting point) in our work are analogous to the one presented in [12,40].

#### 2.3.1. Figure of Merit (FOM)

Focusing light within a specified region inherently involves the optical manipulation of the directivity and intensity of the light. To improve the collection of light in the photosensitive region, the light intensity at a point of interest (POI) needs to be maximized. Therefore, light intensity in a specified region is introduced as FOM. In our study, we aim to maximize the intensity of light at the central wavelengths corresponding to the primary colors. The central wavelengths for the primary colors RGB are at 650 nm, 550 nm, and 450 nm, respectively. The FOM F is given as follows:

(1) $$\;\;\;F={\left|\;E(r)\;\right|}^{2},$$

where E is the electric field at the POI r, located within the Si substrate. The reasons we aim to maximize the FOM in this optimization process are twofold. Firstly, the defined FOM explicitly implies the ability to guide and concentrate light at a specific region, which strongly correlates with the performance of microlenses. Secondly, maximizing the FOM in a specified region located within the substrate ensures the enhanced transmission of light through the Si surface for each pixel.

#### 2.3.2. Optimization Geometry

**Figure 3:**
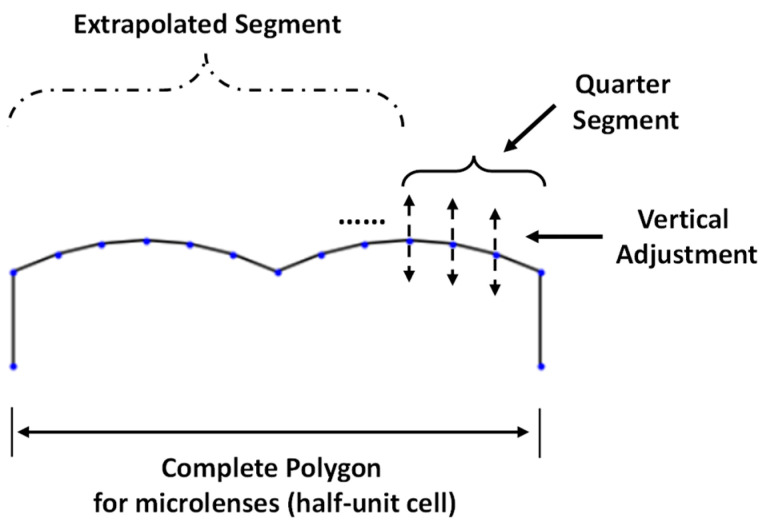
Parameterization of CMOS microlenses showing the cross-sectional view of a half-unit cell Bayer color filter.

For 2D optimization, we parameterized the geometry of CMOS microlenses for a half-unit cell of the Bayer color filter, such as red and green (RG) or green and blue (GB), as previously shown in Figure 1b. A polygon having a limited number of control points is defined to represent the curvature of microlenses. In this work, the control points are considered as the design parameters. A quarter of the parameterized polygon is defined using independent discrete points, as shown in Figure 3. Since the initial design of microlenses is symmetric prior to optimization, we extrapolate the complete geometry from the quarter segment, as shown in Figure 3.

During optimization, the control points are adjusted independently in the vertical direction only, within predefined bounds. This independent adjustment of the design parameters allows the development of asymmetric designs of microlenses. The number of design parameters, i.e., control points, influences the design controllability, manufacturability, and computational cost during optimization. For instance, optimal designs with small minimum feature sizes are usually sophisticated and difficult to realize during the fabrication process. Therefore, shape interpolation [41] among the discrete control points is employed to ensure a smooth boundary at the interface. The utilized interpolation approach reduces oscillation and wiggles in the optimal design by preventing overshooting or undershooting during the optimization process, unlike cubic interpolation [42].

#### 2.3.3. Gradient-Based Optimization

A gradient-based algorithm is used in the optimization framework to maximize the FOM. A quasi-Newton-driven approach, namely the limited-memory Broyden–Fletcher–Goldfarb–Shanno (L-BFGS) algorithm [43,44], is employed to efficiently adjust the design parameters of CMOS microlenses. The design parameters are modified based on the first-order sensitivity, i.e., gradient, and second-order sensitivity, i.e., Hessian, of the FOM. The L-BFGS method determines the direction of search by using both the gradient and Hessian of the FOM. The inverse of the Hessian of the FOM is approximated in a memory-efficient way using this method [44]. Once a search direction is obtained, the step size is calculated by carrying out a line search along that direction. This process is iterative and terminates when the change in the FOM for subsequent iterations falls below a certain threshold. However, as the L-BFGS algorithm is driven by local gradient information, it converges to the local optima in the solution space.

During optimization, the gradient of the FOM with respect to all the design parameters of microlenses is required at every iteration. The gradient of the FOM can be obtained using time-intensive finite difference methods such as forward finite difference (FFD), central finite difference (CFD), and backward finite difference (BFD). The associated computational cost for gradient computation can be prohibitive. In this work, we use ASA as an alternative approach to efficiently compute the gradient of the FOM with respect to all the design parameters of the microlenses.

## 3. Adjoint Sensitivity Analysis (ASA)

ASA calculates the gradient of FOM with respect to all the design parameters using only two field simulations as follows:

1. Forward simulation: The original simulation that includes a physical field driven by an original source.
2. Adjoint simulation: An extra backward simulation that includes a nonphysical field driven by a modified source.

The overhead computational cost for the gradient of the FOM using ASA is effectively insensitive to the number of design parameters. Therefore, adjoint methods are efficient for photonic systems with many design parameters. For the readers’ convenience, this section discusses the general formulation of an adjoint field in the context of nanophotonic device optimization, followed by FDTD-based gradient calculation using ASA.

**Figure 4:**
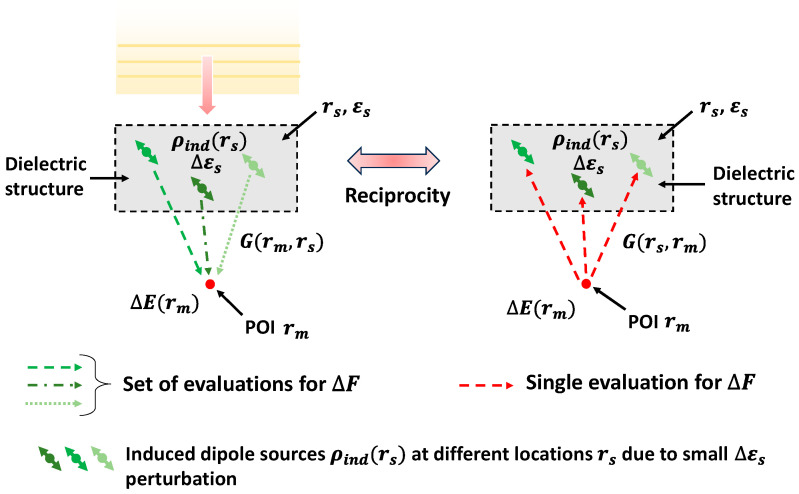
Dipole representation illustrating efficient computation for the change in the FOM ∆F. On the left side, for every $r_{s},$ a separate set of simulations (shown in different types of dotted lines) is required per iteration to compute ∆F,  while by using Green’s reciprocity in the dipole representation, ∆F is evaluated at once using one single simulation for all $r_{s}$ on the right side.

To illustrate the general formulation of an adjoint field, we consider a dielectric structure with permittivity $\varepsilon_{s}\;$illuminated by incident wave, as shown in Figure 4. The goal is to optimize the dielectric structure through a guided perturbation that maximizes the field intensity at an arbitrary neighboring point $r_{m}$. The expression for the FOM can be simply given as follows:

(2) $$F={\left|\;E(r_{m})\;\right|}^{2}=E(r_{m})\cdot \bar{E(r_{m})},$$

where $E\left(r_{m}\right)$ and $\bar{E(r_{m})}$ are the electric field and its complex conjugate, respectively, at the POI $r_{m}$. Any small perturbation $∆\varepsilon_{s}$ in the dielectric structure will induce a change in $E\left(r_{m}\right)$. The change in the FOM due to the small perturbation is given by the following:

(3) $$\Delta F=E^{new}(r_{m})\cdot \bar{E^{new}(r_{m})}-E^{org.}(r_{m})\cdot \bar{E^{org.}(r_{m})},$$

where $E^{org.}$ and $E^{new}\;$denote the electric fields for the original (i.e., unperturbed) and new (i.e., perturbed) approaches, respectively. If the change in the electric field $∆E(r_{m})$ at $r_{m}\;$ is small enough, then by dropping the higher order terms and after some manipulations, Equation (3) can be reduced to the following [29]:

(4) ΔF≈ReEorg.(rm)¯ ΔE(rm)

The dielectric perturbation $∆\varepsilon_{s}\;$of an infinitesimal volume ∆V at an arbitrary point $r_{s}$ in the structure induces polarization density $\rho_{ind}\left(r_{s}\right)$, as illustrated in Figure 4. This acts as a local dipole or point source. The electric field change $∆E\left(r_{m}\right)\;$at $r_{m}\;$ due to the induced dipole located at $r_{s}\;$can be calculated using Green’s function, as presented in the following [29]:

(5) $$\Delta E(r_{m})=G(r_{m}{,r}_{s})\rho_{ind}(r_{s}),$$

where $G(r_{m},r_{s})$ is the Green’s function that relates to the electric field change $∆E\left(r_{m}\right)$ at $r_{m}$ due to the dipole source $\rho_{ind}\;$at $r_{s}$, as shown in Figure 4. By substituting the expression of $∆E\left(r_{m}\right)$ in Equation (4), the change in the FOM ∆F can be written as follows:

(6) ΔF≈ReEorg.(rm) ¯G(rm,rs)ρind(rs)

In essence, for passive, isotropic, and dielectric media, the Green’s function $G(r_{m},r_{s})$ is reciprocal, i.e., $G\left(r_{m},r_{s}\right)=G\left(r_{s},r_{m}\right)$. Therefore, by substituting and rearranging Equation (6), it can be formulated as follows:

(7) ΔF≈ReG(rs,rm)Eorg.(rm)¯ρind(rs)

The term $G(r_{s},r_{m})\bar{E^{org.}\left(r_{m}\right)}$, in Equation (7), is an EM field driven by a dipole source located at $r_{m}\;$with amplitude $\bar{E^{org.}\left(r_{m}\right)}$. This nonphysical EM field is considered as the adjoint field $E^{adj.}(r_{s})$. As the adjoint field $E^{adj.}(r_{s})$ represents a single dipole simulation, it can be computed at once.

**Figure 5:**
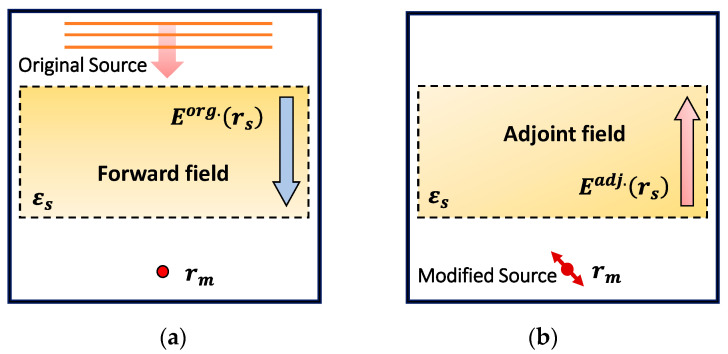
(**a**) Forward field, a physical field, driven by original source; (**b**) adjoint field, a nonphysical field, driven by modified dipole source.

In this work, we have implemented the abovementioned adjoint formulations in FDTD to carry out sensitivity analysis. In the FDTD grid, as shown in Figure 5, the gradient of the FOM F at $r_{m}\;$can be approximated as follows:

(8) $$\frac{dF}{d\varepsilon_{s}}≅E^{\mathrm{org}.}(r_{s})E^{adj.}(r_{s}),$$

where $E^{org.}\left(r_{s}\right)$ and $E^{adj.}(r_{s})$ are the forward and adjoint electric fields at every point $r_{s}$ of the optimizable structure in the FDTD grid, respectively, as shown in Figure 5. It is important to note that only two FDTD simulations are carried out in Equation (8) for each iteration to obtain the first-order sensitivity (or gradient) of the FOM F with respect to the material permittivity $\varepsilon_{s}$.

## 4. Results

To evaluate the functionality and robustness of the optimization approach and the performance of the optimized CMOS microlenses, we analyze the simulation results in several steps. First, we evaluate the baseline functionality of the optimizer by investigating the development of FOM, i.e., the intensity of light in a specified region. This helps to understand how effectively the optimizer improves the focusing of incoming light into the photosensitive region. In the next few steps, the robustness of the optimization process is assessed by evaluating its performance across a wide range of conditions involving the source, optimizable geometry, and focusing region. The performance of the optimized CMOS microlenses is evaluated for light entering at various angles as it mirrors the real-world scenario. The results are compared with the performance studies presented in [40] for the conventional microlenses. Furthermore, we also study 3D optimization to evaluate the optimizer’s ability to identify optimal designs within the design space of higher dimensions and obtain a more accurate physical representation of CMOS microlenses.

### 4.1. Polarization-Specific Optimization

**Figure 6:**
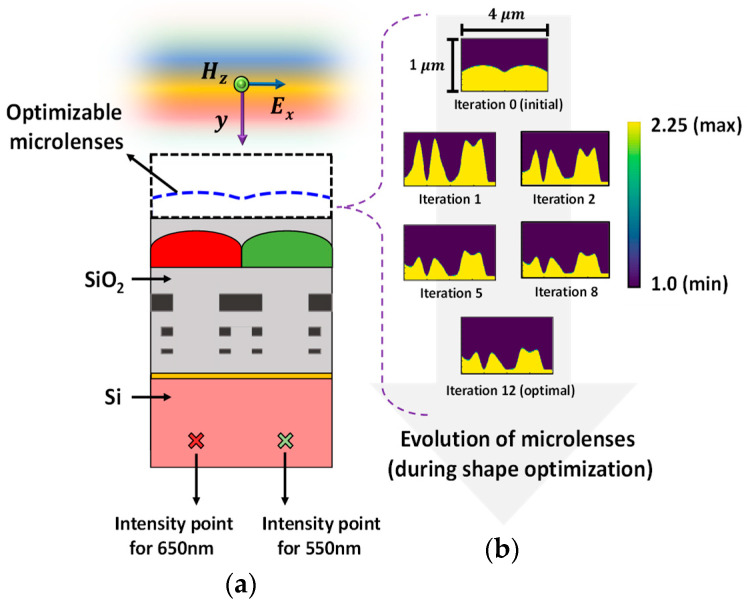
(**a**) Illumination of the TE-polarized plane wave on CMOS image sensor. The optimizable microlenses are shown in the curved dotted line above the RG color pixels. The POIs, to maximize the light intensity at the wavelengths of 550 nm and 650 nm, are shown as ‘×’ in the Si substrate; (**b**) the evolution of the optimal CMOS microlenses during the optimization process.

The optimization process is carried out for both polarization of the source: TE polarization and TM polarization. The optimization of microlenses is implemented for a half-unit cell of the Bayer color filter to enhance FOM at a specific point. The microlenses for red and green (RG) color pixels are optimized simultaneously for two different POIs at 650 nm and 550 nm wavelength, respectively, as shown in Figure 6. TE-polarized source condition is used in this case. The two selected POIs (indicated as ‘×’ in Figure 6a), located under the RG color pixels, are spatially separated and positioned approximately 60% of the depth within the 1.2 μm thick Si substrate. The optimizable microlenses are confined within a region with a dimension of 1 μm × 4 μm. The permittivity distribution of CMOS microlenses over the optimization process at different steps is illustrated in Figure 6b. The permittivity used for air and microlenses are 1.0 and 2.25, respectively. Figure 6b depicts that after several rounds of iterations, the optimal design of microlenses that maximizes the intensity of light at the POIs corresponding to two different wavelengths is obtained under TE-polarized condition.

**Figure 7:**
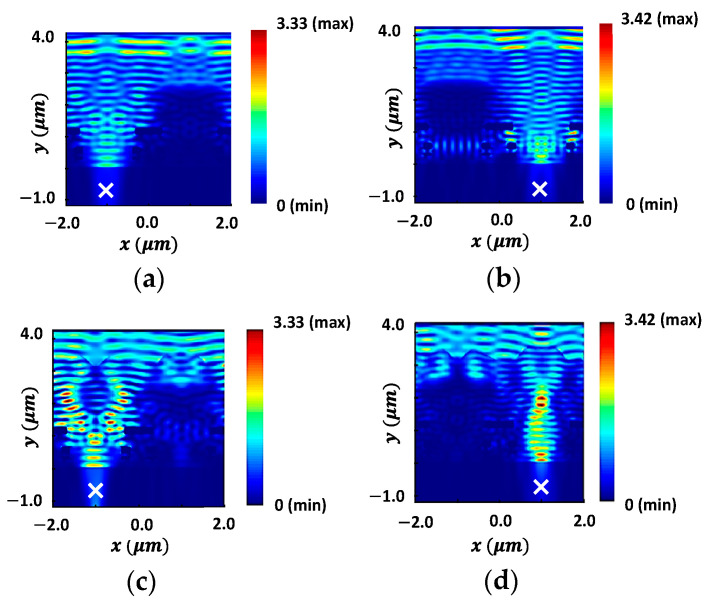
Distribution of field intensity for the initial design of microlenses at the wavelengths of (**a**) 650 nm and (**b**) 550 nm. The POIs, to maximize the light intensity, are shown as ‘×’ in the substrate; the distribution of field intensity for the optimal design of microlenses at the wavelengths of (**c**) 650 nm and (**d**) 550 nm.

To demonstrate the improvement in light focusing, the field intensity distributions for the initial design of microlenses, prior to optimization, at the wavelengths of 650 nm and 550 nm are depicted in Figure 7a,b, respectively. Figure 7c,d illustrate the distributions of field intensity corresponding to the optimized microlenses. The POI is indicated as ‘×’ in Figure 7, where the light intensity is aimed to be maximized. In contrast to Figure 7a, an enhanced transmission of light at a wavelength of 650 nm through the red color pixel is observed in Figure 7c. Similarly, it is evident that the transmission of light at 550 nm wavelength through the green color pixel, in Figure 7d, is significantly higher and focused as compared to Figure 7b.

**Figure 8:**
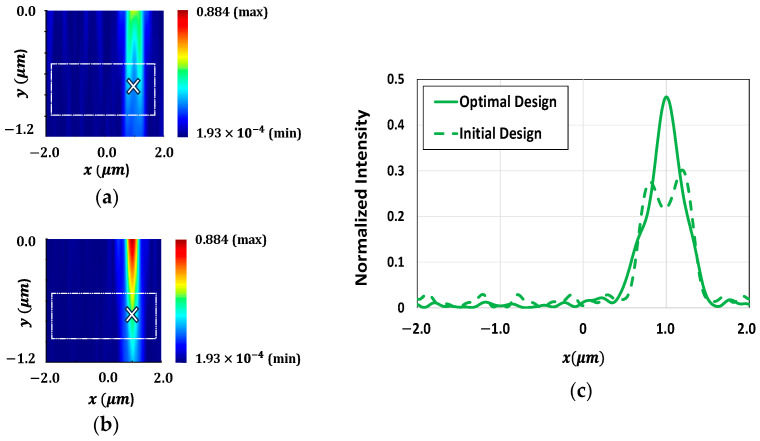
Distribution of field intensity surrounding the POI (marked as ‘×’) within the Si substrate for green color pixel at a wavelength of 550 nm for the (**a**) initial design and (**b**) optimal design of microlenses; (**c**) light intensity at the POI for both the initial and optimal configurations of microlenses at 550 nm wavelength.

**Figure 9:**
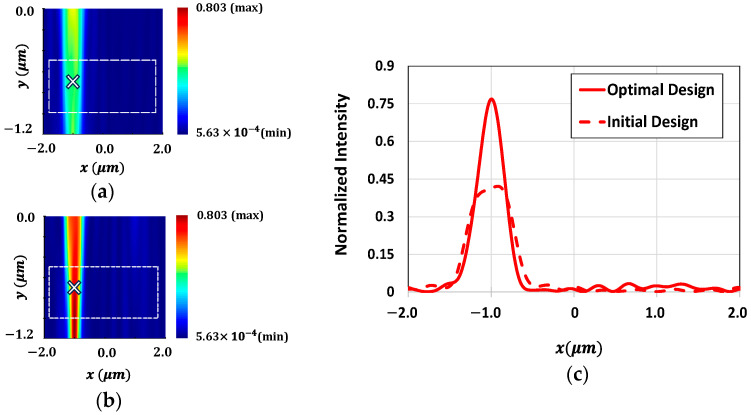
Distribution of field intensity surrounding the POI (marked as ‘×’) within the Si substrate for red color pixel at a wavelength of 650 nm for the (**a**) initial design and (**b**) optimal design of microlenses; (**c**) light intensity at the POI for both the initial and optimal configurations of microlenses at 650 nm wavelength.

The concentration of light around the POIs within the Si substrate is further illustrated in Figure 8. Figure 8a,b show the intensity of light at 550 nm wavelength around the POI (indicated with an ‘×’ in the figure) within the Si substrate for the initial and optimal design of microlenses, respectively. Figure 8b clearly shows that the light intensity near the POI for the optimized microlenses is significantly enhanced as compared to the light intensity observed for the initial design, as shown in Figure 8a. Figure 8c presents the intensity of light at 550 nm wavelength for both configurations at the POI. Figure 9a,b demonstrate the focusing of light at a wavelength of 650 nm near the POI for both before and after optimization, respectively. Figure 9b exhibits a strong focusing of light at a wavelength of 650 nm near the POI compared to the initial design, as shown in Figure 9a. The light intensity at the POI for both designs, before and after optimization, at 650 nm wavelength are depicted in Figure 9c. It shows that the microlenses exhibit nearly 50% enhancement in light intensity after optimization.

**Figure 10:**
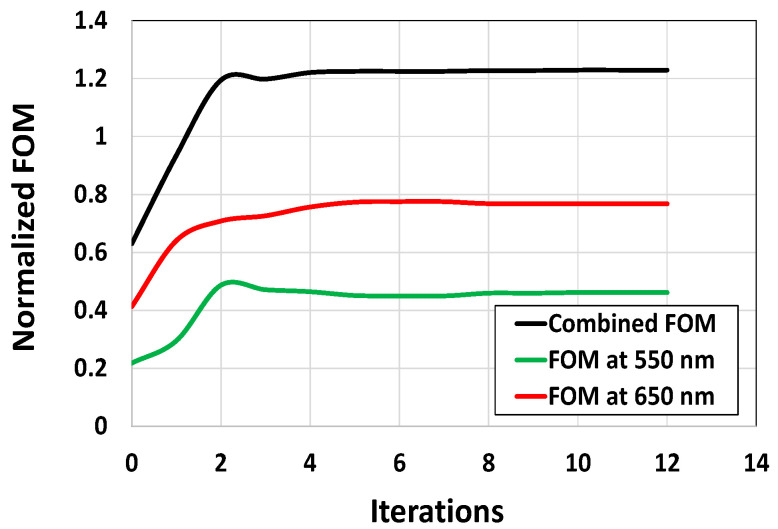
The progression of the figures of merit (FOMs) over the optimization of CMOS microlenses.

Figure 10 presents the progression of the FOMs over the iterative optimization process. While the FOMs for both wavelengths, i.e., 550 nm and 650 nm, change significantly within the first few iterations, the optimization process converges after the 12th iteration. This suggests that by leveraging the gradient information, the optimizer successfully reached an optimal design, as there is no significant change in the FOM development across consecutive iterations. All the results obtained from Figure 6 to Figure 10 collectively indicate that the optimal design of CMOS microlenses for the RG color pixels developed by an adjoint-driven optimizer efficiently focuses and enhances the intensity of the incoming light at POI.

**Figure 11:**
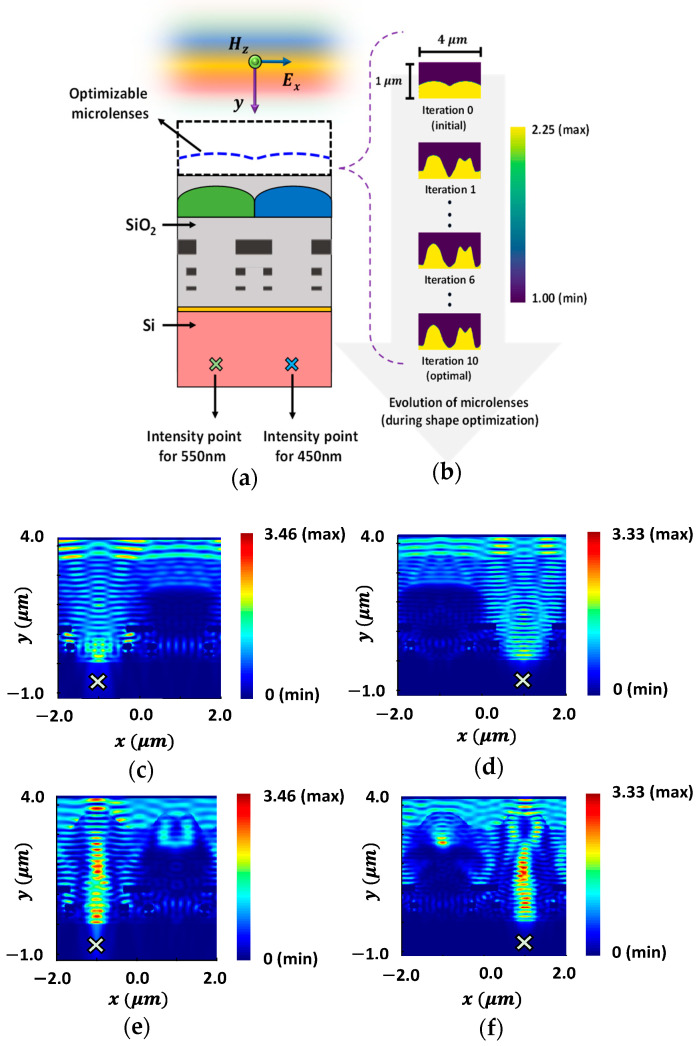
(**a**) Illumination of the TE-polarized plane wave on CMOS image sensor. The optimizable microlenses are shown in the curved dotted line above the GB color pixels. The POIs, to maximize the light intensity at the wavelength of 450 nm and 550 nm, are shown as ‘×’ in the Si substrate; (**b**) the evolution of the optimal microlenses during the optimization process; the distribution of field intensity for the initial design of microlenses at the wavelengths of (**c**) 550 nm and (**d**) 450 nm; the distribution of field intensity for the optimal design of microlenses at the wavelengths of (**e**) 550 nm and (**f**) 450 nm.

Afterwards, we performed the 2D optimization of CMOS microlenses for the other set of half-unit cell of the Bayer color filter. We optimized the microlenses for green and blue (GB) color pixels to maximize the light intensity at the POIs for two different wavelengths, i.e., 550 nm and 450 nm. The optimization results are illustrated in Figure 11. A TE-polarized plane wave excites the CMOS structure, as illustrated in Figure 11a. We employed a similar parameterization, using adjustable control points, to define the surface curvature of the microlenses. Figure 11b shows the evolution of microlenses through the iterative changes in the design parameters. The distributions of field intensity corresponding to the initial design of microlenses for GB color pixels at the wavelengths of 550 nm and 450 nm are depicted in Figure 11c,d, respectively. Similarly, Figure 11e,f illustrate the field intensity distributions for the optimized microlenses at 550 nm and 450 nm wavelengths, respectively. The results presented in Figure 11e,f suggest an improved focusing and enhancement in the field intensity of the incoming light after optimization.

**Figure 12:**
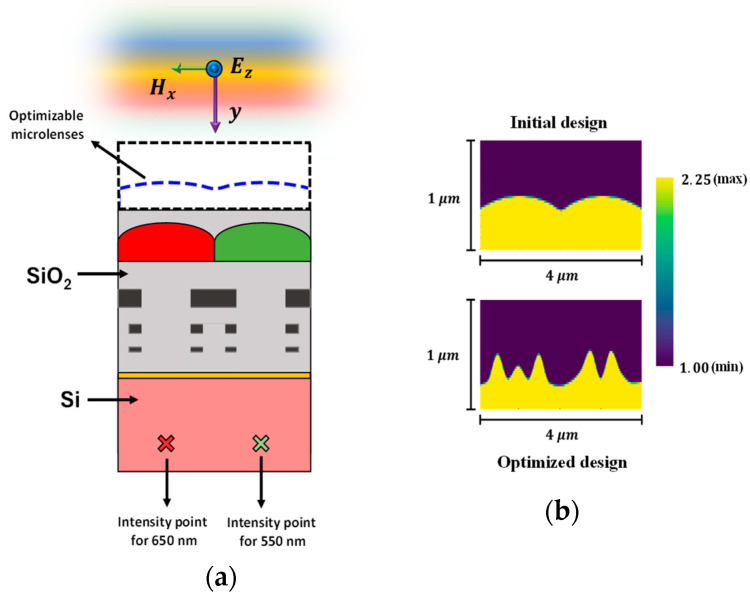

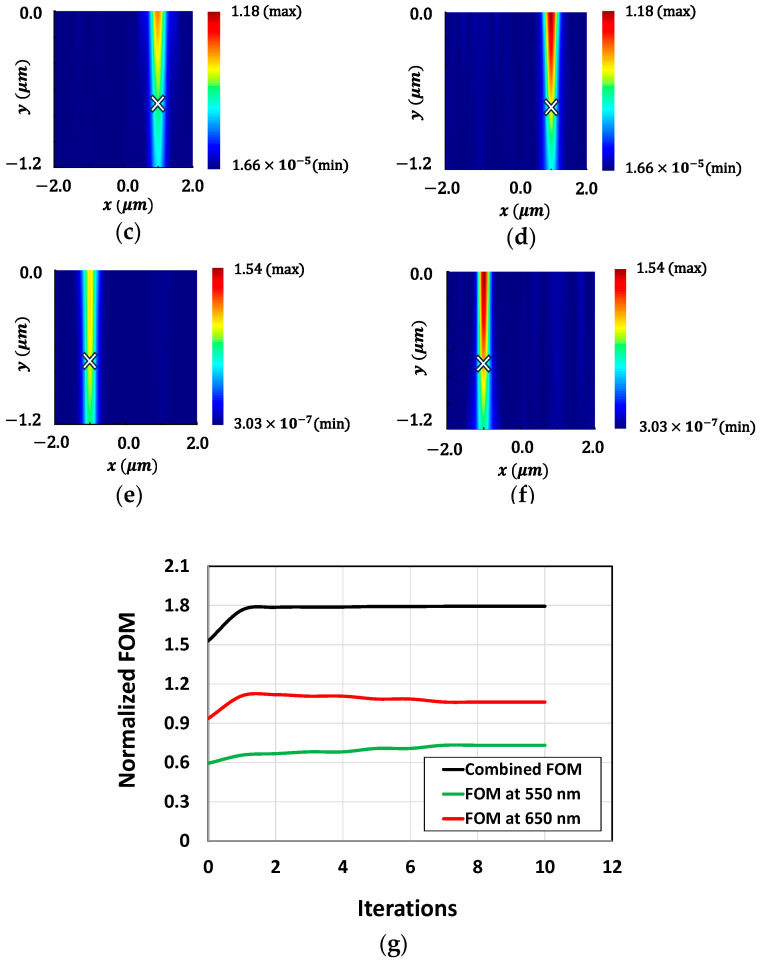
(**a**) Illumination of the TM-polarized plane wave on CMOS image sensor. The optimizable microlenses are shown in the curved dotted line above the RG color pixels. The POIs, to maximize the light intensity at the wavelength of 550 nm and 650 nm, are shown as ‘×’ in the Si substrate; (**b**) the design of CMOS microlenses for the RG color pixels before and after optimization under TM polarization; the distribution of field intensity surrounding the POI (marked as ‘×’) under TM-polarized light at a wavelength of 550 nm for (**c**) initial design and (**d**) optimal design; the distribution of field intensity surrounding the POI (marked as ‘×’) under the illumination of TM-polarized light at a wavelength of 650 nm for (**e**) initial design and (**f**) optimal design; (**g**) the progression of FOMs during the optimization process for RG color pixels under TM-polarized source condition.

The optimization process is also carried out for TM-polarized source conditions, optimizing the microlenses for the RG color pixels. A TM-polarized plane wave illuminates the CMOS microlenses for the RG color pixels, as illustrated in Figure 12a. The microlenses before and after optimization are shown in Figure 12b. Figure 12c,d present the light intensity distributions at 550 nm wavelength corresponding to the initial and optimal design of microlenses, respectively. Similar distributions for the initial and optimal designs at 650 nm wavelength are shown in Figure 12e,f, respectively. The results suggest that the optimized design exhibits a strong focusing of light along with enhanced intensity around the POI for both wavelengths. The development of FOMs throughout the optimization process is depicted in Figure 12g. It shows that the combined FOM improves for the first few iterations and converges after the 10th iteration.

**Figure 13:**
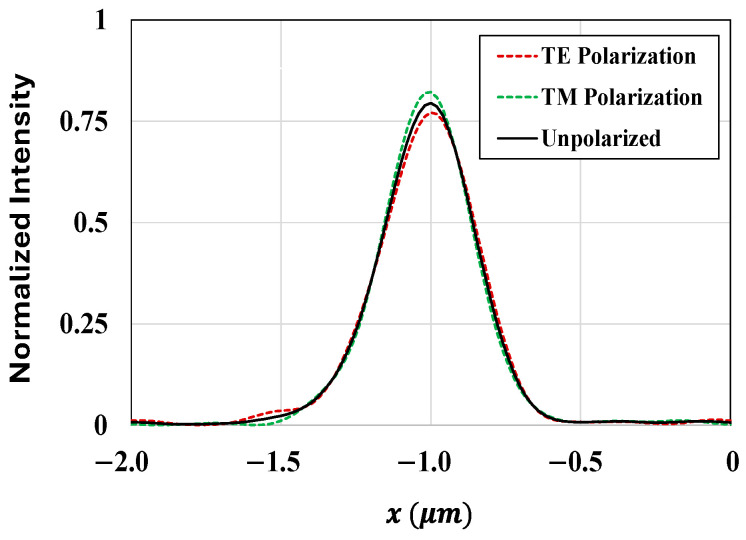
Field intensity at the POI for the optimized microlenses corresponding to the RG color pixels at 650 nm wavelength under various polarizations.

Furthermore, to ensure that the optimized structure exhibits robust and consistent behavior in real-world scenarios, we evaluated the performance of the optimized CMOS microlenses under unpolarized light conditions. The field intensity at the POI for the optimized microlenses corresponding to the RG color pixels at 650 nm wavelength for unpolarized light is illustrated in Figure 13. The performance for unpolarized light is determined by evaluating the responses of the optimal microlenses for both the TE and TM polarizations separately.

### 4.2. Incident Angles

**Figure 14:**
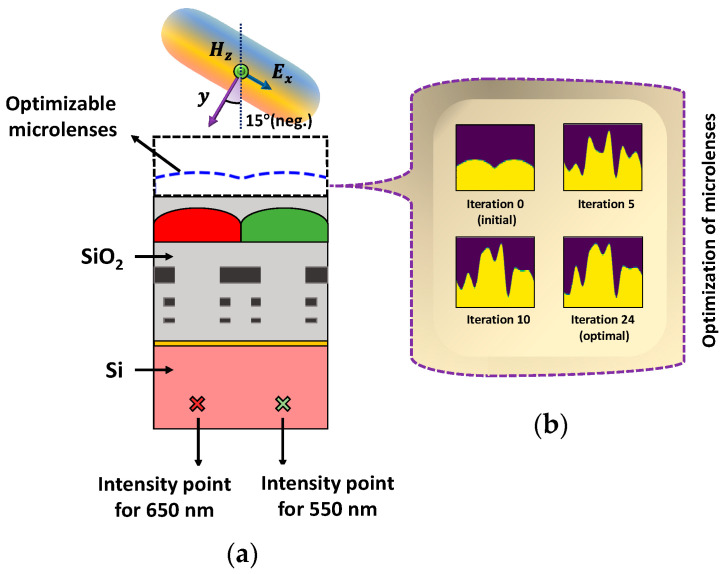

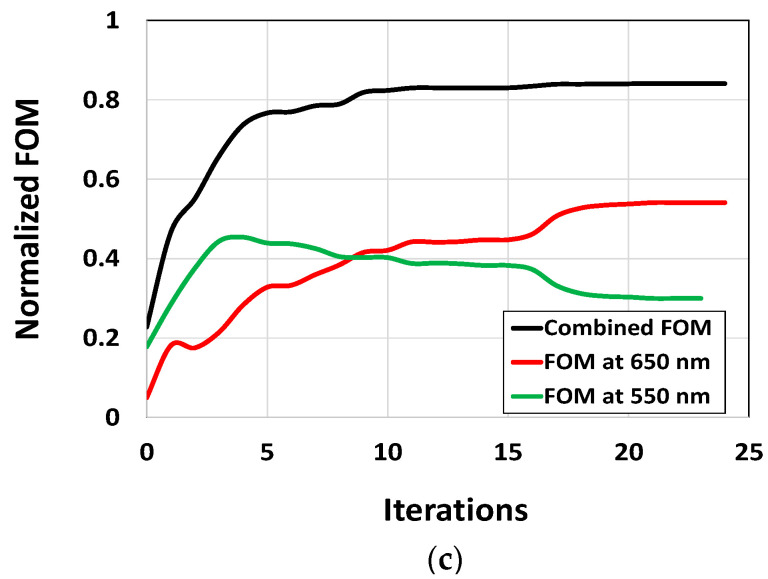
(**a**) Illumination of the TE-polarized plane wave on CMOS image sensor with an oblique incident angle (i.e., −15°). The optimizable microlenses are shown in the curved dotted line above the RG color pixels. The POIs, to maximize the light intensity at 550 nm and 650 nm wavelengths, are shown as ‘×’ in the Si substrate; (**b**) the evolution of optimal designs for CMOS microlenses during the optimization process for oblique incident angle; (**c**) the development of FOMs over the iterations for a tilted incident angle.

Variation in incident angles is also considered to evaluate the performance of the optimizer. We carried out the optimization of CMOS microlenses for oblique incident light. The optimization process is studied for TE-polarized illumination with an oblique incident angle of 15°, as indicated in Figure 14a. Figure 14b briefly depicts the evolution of the optimized microlenses corresponding to the RG color pixels. The progression of FOMs for the combined and individual color pixels is illustrated in Figure 14c. Figure 14c suggests that while the optimizer attempts to improve the FOM for the red color pixel at 650 nm wavelength over the optimization period, the FOM corresponding to the green color pixel at 550 nm wavelength gradually deteriorates after a few rounds of iterations. This inherent trade-off occurs because the optimizer tries to improve a single, yet conflicting, target at two different wavelengths. Nevertheless, the overall FOM for the RG color pixels is significantly improved. The results illustrated in Figure 14 highlight the ability of the optimizer to focus incoming light with a tilted angle at the POI.

### 4.3. Parameterization

**Figure 15:**
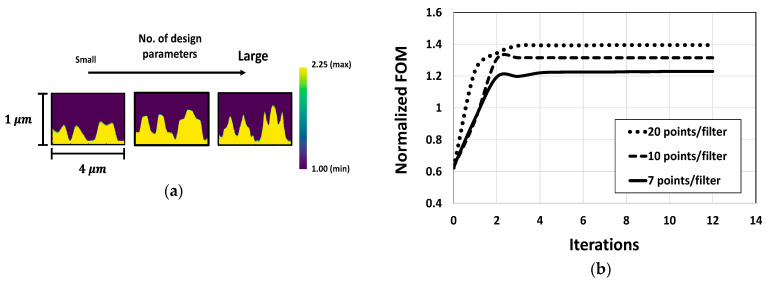
(**a**) Optimal designs of CMOS microlenses for different parameterization; (**b**) the evolution of FOMs corresponding to the different parameterization of CMOS microlenses.

The optimization approach is also explored for CMOS microlenses through variation in the parameterization. For this purpose, we evaluate the performance of the optimized microlenses by varying the number of control points. While design parameters in a large number lead us to obtain optimal designs with slightly enhanced performance, this often increases design complexity, as illustrated in Figure 15a,b. Furthermore, the variation in FOMs across the optimized designs with different parameterizations is observed to be relatively small, ranging from 1.2 to 1.4. This suggests that the optimizer can find different optimized structures depending on the initial design without significant deviation in the FOMs.

### 4.4. Region of Interest (ROI)

**Figure 16:**
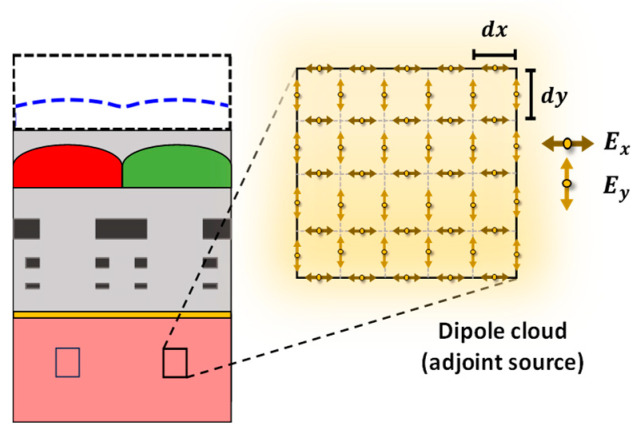
The spatial arrangement of the adjoint field driven by multiple dipole sources (or dipole cloud) in staggered FDTD to maximize the FOM over the AOI.

In all the previous experiments, we only considered maximizing the light intensity at a specific point or POI. However, maximizing the light intensity over an area is often preferable for real-life applications. This allows for capturing more of the incoming light, leading to an improved image quality. Therefore, the microlenses are further optimized to improve the FOM over a specified area instead of a point. It is important to highlight that previously, the dipole sources were placed at the POIs to drive the adjoint FDTD simulations. Once we attempt to optimize the design for an area of interest (AOI), the spatial arrangement for adjoint dipole sources is slightly different due to the staggered nature of the FDTD grid. The arrangement for adjoint dipole sources in the staggered FDTD is presented in Figure 16. Such an arrangement of adjoint dipole sources represents a cloud of dipoles.

**Figure 17:**
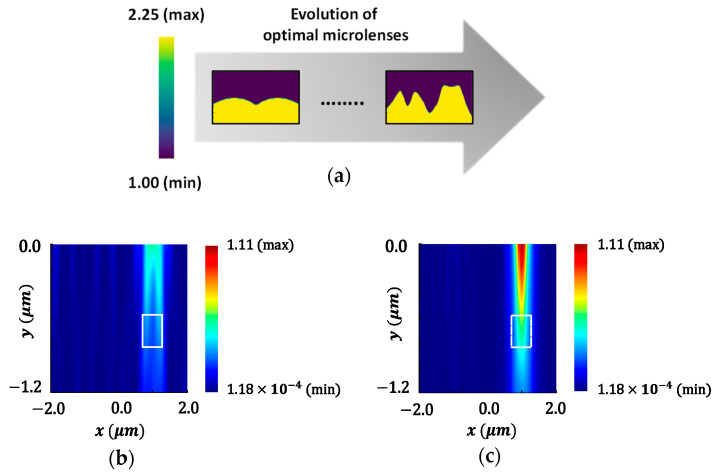

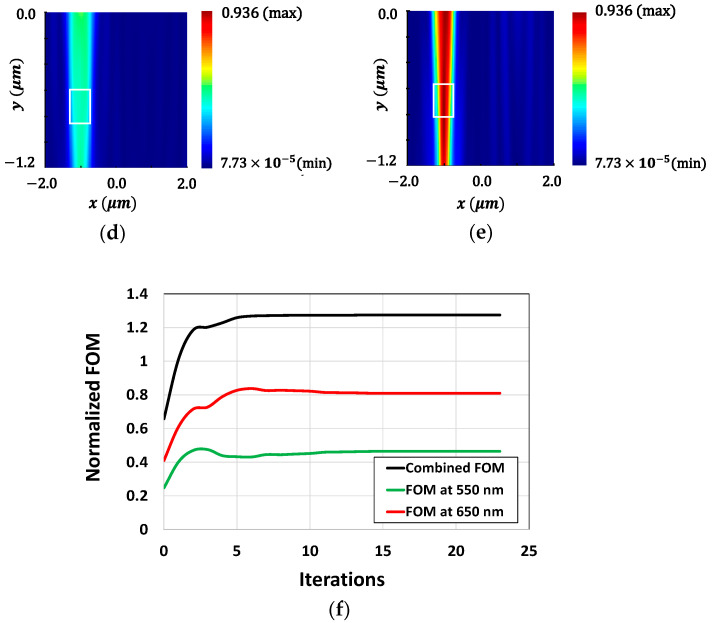
(**a**) Evolution of the optimal microlenses to maximize the intensity over the AOI (shown as the dotted rectangular box) under TE polarization; the distribution of field intensity at a wavelength of 550 nm for the (**b**) initial and (**c**) optimal configurations; the distribution of field intensity at a wavelength of 650 nm for the (**d**) initial and (**e**) optimal design; (**f**) the progression of FOMs to improve focusing over the AOI throughout the optimization process.

Figure 17 demonstrates the optimization results of CMOS microlenses for the RG color pixels, showing that the light intensity is maximized over a specific area. TE-polarized excitation is used in this case. The gradual evolution of the optimal microlenses with enhanced light intensity over the AOI is shown in Figure 17a. The light intensity distribution over the specified AOI (indicated as the dotted rectangular box) for both the initial and optimized design at 550 nm wavelength are shown in Figure 17b,c, respectively. Figure 17d,e demonstrate the distribution of field intensity at a wavelength of 650 nm corresponding to the initial and optimized configurations of microlenses, respectively. The results suggest that the focusing and intensity of the incoming light are significantly enhanced for the optimized microlenses in the RG color pixels. The iterative improvements in FOMs for the AOI during the optimization process are illustrated in Figure 17f. It shows that the combined FOM at both wavelengths gradually increases over the optimization period and the optimal design is achieved after 24 iterations. The comprehensive studies and results presented in Figure 6 to Figure 17, covering the FOM developments under various source conditions, parameterizations, and focusing regions, collectively validate the fundamental functionality and robustness of the adjoint-assisted optimization framework.

### 4.5. Optical Efficiency (OE)

Following the comprehensive evaluation of the optimizer’s base functionality and robustness, the performance of the optimized CMOS microlenses is compared against the conventional microlenses, presented in [40]. The performance between the conventional and optimized microlenses is compared using the angular response (AR). The AR measures the OE of the device with variation in the incident angle. One of the primary reasons for using AR as a performance metric is that it reflects how efficiently the CMOS microlenses can focus the light arriving from different angles simultaneously. This ensures a consistent image quality despite light entering at steeper angles.

The performance evaluation is carried out in two steps. First, we systematically optimize the CMOS microlenses for the RG color pixels for light entering at inclined angles, ranging from −10° to +10°. This is important to highlight that the initial design used in the optimization process is analogous to the conventional design of microlenses reported in [12,40]. In the second step, we compute the OE for each color pixel using the formulation as follows [45]:

(9) $$OE=\frac{P_{abs}(i)}{P_{inc}}$$

where $P_{abs}\left(i\right)$ corresponds to the power absorbed on the Si surface for the $i^{th}$color pixel, and $P_{inc}$ represents the power of the incident light.

**Figure 18:**
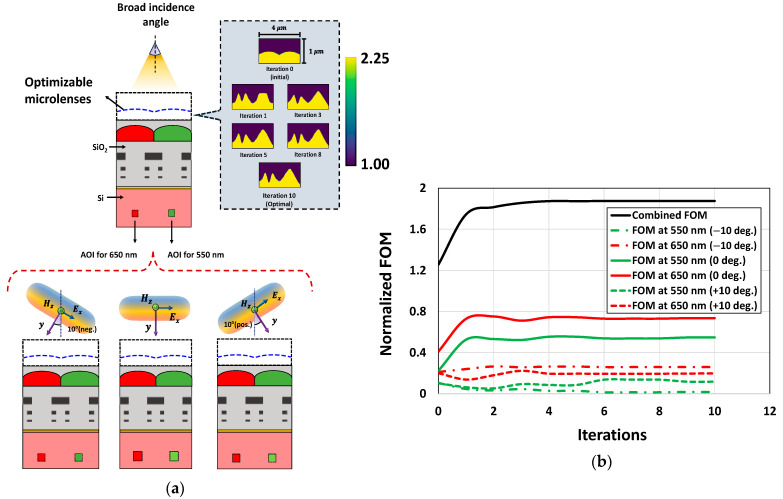
(**a**) The iterative development of the optimal CMOS microlenses for the RG color pixels during the optimization process. The microlenses are optimized for light entering at an oblique angle. The incident angles are ranging from −10° to +10°. Photonic setups for light entering at three different angles, i.e.,−10°, 0°, and +10°, are shown at the bottom; (**b**) the progression of the FOMs across multiple incident angles at the wavelengths of 650 nm and 550 nm.

To carry out the optimization process, we optimize the CMOS microlenses for incident light coming at three different selective angles, i.e., −10°, 0°, and +10°, as shown (at the bottom) in Figure 18a. The optimizer attempts to find an optimal design that improves the FOMs at these incident angles. Figure 18a shows the gradual development of the optimal design of CMOS microlenses which simultaneously improves the FOMs for three different photonic setups. The optimization process is carried out concurrently for all three photonic setups, having different incident angles. The optimizer attempts to improve the overall FOM over a specified AOI. The progression of FOMs across three different angles for the RG color pixels at the wavelengths of 550 nm and 650 nm is depicted in Figure 18b. Figure 18b indicates that the combined FOM enhances gradually over the first few iterations and converges after 10 iterations. It is also observed that the FOMs corresponding to the RG color pixels significantly enhance at a normal incident angle. While finding a balance among the FOMs corresponding to different incident angles, an improvement is observed in the FOMs for the wavelengths of 550 nm and 650 nm at incident angles of +10° and −10°, respectively. This suggests that the optimized microlenses improved the focusing of light at the AOI for the green and red color pixels at an incident angle of +10° and −10°, respectively. However, slight deterioration in the FOMs is noticed during the optimization process for the wavelengths of 550 nm and 650 nm at the incident angles of −10° and +10°, respectively. The inadvertent reduction in FOMs at certain angles can be expected as the optimizer attempts to improve a single FOM across multiple angles simultaneously.

Once the design of microlenses is optimized through adjoint-driven optimization, a parametric sweep is carried out to calculate the OE of the optimal design for unpolarized light. The OE for unpolarized light is determined by averaging OE for both the TE and TM polarizations. We use the expression in Equation (9) to obtain the OE corresponding to the RG color pixels. The power absorbed $P_{abs}\;$ is determined by integrating the Poynting vector over a specified surface of the Si substrate for the respective color pixels. The specified surface is centered beneath each color pixel, having a width of 1 μm. To determine the AR, the OE of the optimal design is evaluated across various incident angles, ranging from −10° to +10°, using a nested parametric sweep. Both the TE and TM polarizations are considered at each angle, leading to a total of 42 FDTD simulations to evaluate the AR of the optimized microlenses.

**Figure 19:**
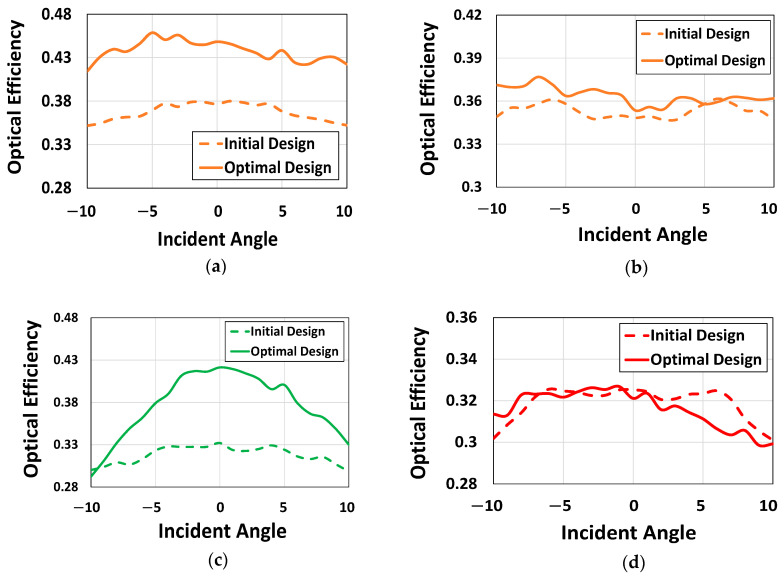
Optical efficiency corresponding to the initial and optimal configurations for unpolarized light at the Si surface for (**a**) 550 nm and (**b**) 650 nm wavelength; optical efficiency corresponding to the initial and optimal configurations for unpolarized light at the Si surface beneath the (**c**) green and (**d**) red color pixel.

The OE for CMOS microlenses, having RG color pixels, are depicted in Figure 19. For unpolarized light, the AR corresponding to both the designs, initial and optimal, at the wavelengths of 550 nm and 650 nm are illustrated in Figure 19a,b, respectively. Figure 19a clearly shows that the optimized design achieves substantial improvement across a wide range of incident angles at a wavelength of 550 nm, surpassing the optical performance of both the initial and conventional designs reported in [40]. We further investigated the optical performance of the optimized microlenses at 650 nm wavelength for unpolarized conditions. Figure 19b suggests that the optimized microlenses exhibit better optical response at 650 nm wavelength nearly across all the incident angles in contrast to the initial design. The AR corresponding to the green and red color pixels of the microlenses are illustrated in Figure 19c,d, respectively. It is evident from Figure 19c that the green color pixel of the optimized design demonstrates significantly higher OE for unpolarized light compared to both the initial and conventional microlenses presented in [40]. While the green color pixel shows improved performance for positively inclined angles, the AR of the red color pixel, as shown in Figure 19d, gradually deteriorates for the same set of angles. Nevertheless, the red color pixel shows relatively enhanced performance for unpolarized light at a negatively inclined incident angle in contrast to the initial design.

### 4.6. Optimization in 3D

**Figure 20:**
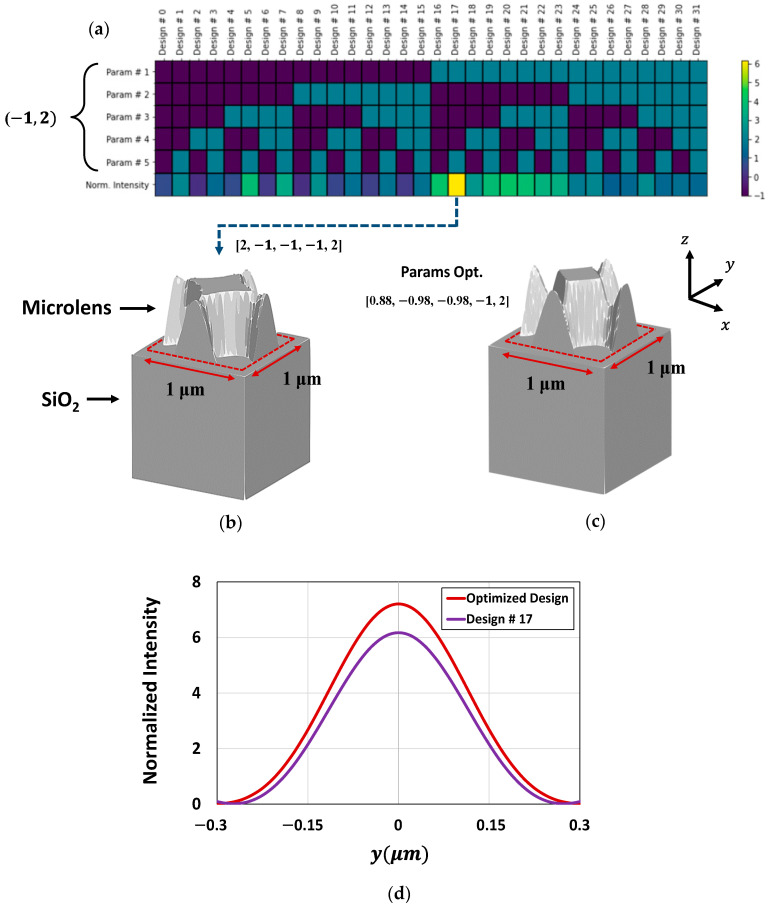
(**a**) Heatmap of 32 distinct microlens designs with normalized intensity; (**b**) 3D view of the best design, i.e., design # 17, obtained through parametric sweep in the design space; (**c**) 3D view of the optimized design obtained through the adjoint-driven optimization approach; (**d**) normalized intensity at the POI for both design # 17 and the optimized design of the 3D microlenses.

Optimization for the design of CMOS microlenses is further carried out in 3D to study how effectively the adjoint-based optimizer can identify the most efficient design in the design space. Additionally, 3D optimization offers a more accurate physical representation of the device geometry, ensuring the most efficient design is better suited for real-world applications. However, the computational resources and time required to perform a full-scale 3D optimization for CMOS microlenses are substantially significant. Therefore, we limit our focus to the microlens design for only a quarter of the unit cell of Bayer color filters by leveraging the inherent symmetry and repetitive arrangement of the CMOS microlenses. Furthermore, the optimization of 3D microlens is carried out to collect the incoming light for a reduced pixel size of 1 μm. A simple CMOS structure containing a Si substrate covered by a microlens, as shown in Figure 20, is used to optimize the usage of computational resources.

In this study, we use a different parameterization using the Fourier basis function to describe the smooth geometric shape of 3D microlens. Geometric parameterization using the Fourier basis functions with appropriate conditions allows structural symmetry and uniform variation in CMOS microlenses during optimization. The number of Fourier coefficients for each spatial dimension is carefully selected to ensure that the expansion covers a consistent order across all the dimensions. These coefficients represent the design parameters that control the geometric features of the 3D microlens. Five design parameters are used as Fourier basis coefficients to perturb the surface curvature of the microlens. To limit the number of possible combinations of microlens designs in the design space, we allowed only two discrete boundary values (−1 and 2) for each design parameter. This results in 32 distinct microlens designs within the design space. It is important to highlight that the number of design combinations in the design space is deliberately constrained by using five design parameters so that the ability of the optimizer to find an optimal design can be closely examined. Using a large number of design parameters (or Fourier basis coefficients) will produce a large design space that is computationally exhaustive. Alternatively, having a small number of design parameters results in a small design space which does not allow us to adequately evaluate the ability of the optimizer.

The light intensity at the POI across all 32 different 3D microlens is obtained through a parametric sweep and represented as a heatmap in Figure 20a. Among the design combinations, design # 17 appears to be the best possible design, demonstrating the highest light intensity at the POI for an optimal set of design parameters (or coefficients), as depicted in Figure 20a. The 3D microlens structure corresponding to design # 17 is depicted in Figure 20b. To investigate the ability of the adjoint-optimizer to find the optimal design of 3D microlens, optimization in 3D is carried out. Figure 20c presents the optimized 3D microlens structure we achieved after adjoint-assisted 3D optimization. During the optimization process, the five design parameters are adjusted between the two discrete bounds (−1, 2). The optimal set of design parameters (or coefficients) attained by the optimization framework is close to those in design # 17. The light intensity at the POI for both the optimized microlens and design # 17 are presented in Figure 20d. Figure 20d shows that the most efficient design of 3D microlens identified by the optimizer exhibits enhanced light intensity (almost 17% enhancement) in contrast to design # 17. This study strongly suggests that the developed adjoint-based optimizer can identify the most efficient design in the design space, surpassing the optical performance of the best design, i.e., design # 17, which is identified through an exhaustive parametric sweep.

## 5. Conclusions

This paper presents a systematic and efficient approach leveraging ASA to optimize the shape of the CMOS microlenses. A novel FOM is developed and integrated into the adjoint-assisted optimization framework to improve the focusing and collection of light in the photosensitive region of the CMOS image sensors. A comprehensive evaluation is performed to assess the ability and robustness of the optimizer through variations in source condition, geometric parameterization, and focusing region. Considering the real-life scenarios, the optical performance of the optimized microlenses is measured through AR and compared to the conventional designs reported previously. Furthermore, we carried out 3D optimization to examine the ability of the adjoint-based optimizer to find the most efficient design within the design space. The key findings of our study demonstrate that significant FOM improvement is achieved by optimizing the shape of CMOS microlenses using an adjoint-driven optimization framework. The optimized design of microlenses exhibits improved AR when compared to the existing conventional microlenses. The adjoint-assisted optimization framework is also able to identify the most efficient design from the design space and accelerate the development of a 3D configuration that is potentially well suited for real-life applications. The applicability of the adjoint-based optimization framework using the novel FOM presented in this paper is not only limited to the efficient development of CMOS microlenses. It also can be employed to free space photonic problems and accelerate the systematic and efficient development of optical and photonic devices that require focusing, such as solar concentrators in photovoltaics.

## Data Availability

Data presented in this research work are not publicly available at this moment but can be obtained from the authors upon reasonable request.

## References

[1] Zhang F., Zhang J., Yang C., Zhang X. (2010). Performance Simulation and Architecture Optimization for CMOS Image Sensor Pixels Scaling Down to 1.0 μm. IEEE Trans. Electron Devices.

[2] Bigas M., Cabruja E., Forest J., Salvi J. (2006). Review of CMOS Image Sensors. Microelectron. J..

[3] Rhodes H., Agranov G., Hong C., Boettiger U., Mauritzson R., Ladd J., Karasev I., McKee J., Jenkins E., Quinlin W. CMOS Imager Technology Shrinks and Image Performance. Proceedings of the 2004 IEEE Workshop on Microelectronics and Electron Devices.

[4] McCleary B. (2005). Cross-Talk Correction Methodology for Color CMOS Imagers. Proceedings of the Digital Photography.

[5] Fossati C., Gagliano O., Commandre M., Dunne B. (2005). Microlens Design for CMOS Image Sensor. Proceedings of the Optical Design and Engineering II.

[6] Fesenmaier C.C., Huo Y., Catrysse P.B. (2008). Optical Confinement Methods for Continued Scaling of CMOS Image Sensor Pixels. Opt. Express.

[7] Anzagira L., Fossum E.R. (2015). Color Filter Array Patterns for Small-Pixel Image Sensors with Substantial Cross Talk. J. Opt. Soc. Am. A.

[8] Agranov G., Berezin V., Tsai R.H. (2003). Crosstalk and Microlens Study in a Color CMOS Image Sensor. IEEE Trans. Electron Devices.

[9] Vaillant J., Hirigoyen F. (2004). Optical Simulation for CMOS Imager Microlens Optimization. Proceedings of the Optical Sensing.

[10] Zhang R., Lai L. (2016). Optical Design of Microlens Array for CMOS Image Sensors. Proceedings of the 8th International Symposium on Advanced Optical Manufacturing and Testing Technologies: Desgin, Manufacturing, and Testing of Micro-and Nano-Optical Devices and Systems, and Smart Structures and Materials.

[11] Hirigoyen F., Crocherie A., Vaillant J.M., Cazaux Y. (2008). FDTD-Based Optical Simulations Methodology for CMOS Image Sensors Pixels Architecture and Process Optimization. Proceedings of the Sensors, Cameras, and Systems for Industrial/Scientific Applications IX.

[12] Crocherie A., Vaillant J., Hirigoyen F. (2008). Three-Dimensional Broadband FDTD Optical Simulations of CMOS Image Sensor. Proceedings of the Optical Design and Engineering III.

[13] Huo Y., Fesenmaier C.C., Catrysse P.B. (2010). Microlens Performance Limits in Sub-2 μm Pixel CMOS Image Sensors. Opt. Express.

[14] Nikolova N.K., Bandler J.W., Bakr M.H. (2004). Adjoint Techniques for Sensitivity Analysis in High-Frequency Structure CAD. IEEE Trans. Microw. Theory Tech..

[15] Georgieva N.K., Glavic S., Bakr M.H., Bandler J.W. (2002). Feasible Adjoint Sensitivity Technique for EM Design Optimization. IEEE Trans. Microw. Theory Tech..

[16] Nikolova N.K., Safian R., Soliman E.A., Bakr M.H., Bandler J.W. (2004). Accelerated Gradient Based Optimization Using Adjoint Sensitivities. IEEE Trans. Antennas Propag..

[17] Zhang Y., Ahmed O.S., Bakr M.H. (2014). Wideband FDTD-Based Adjoint Sensitivity Analysis of Dispersive Electromagnetic Structures. IEEE Trans. Microw. Theory Tech..

[18] Bakr M.H., Elsherbeni A., Demir V. (2017). Adjoint Sensitivity Analysis of High Frequency Structures with MATLAB.

[19] Ghassemi M., Bakr M., Sangary N. (2013). Antenna Design Exploiting Adjoint Sensitivity-based Geometry Evolution. IET Microw. Antennas Propag..

[20] Koziel S., Mosler F., Reitzinger S., Thoma P. (2012). Robust Microwave Design Optimization Using Adjoint Sensitivity and Trust Regions. Int. J. RF Microw. Comput. Eng..

[21] Lalau-Keraly C.M., Bhargava S., Miller O.D., Yablonovitch E. (2013). Adjoint Shape Optimization Applied to Electromagnetic Design. Opt. Express.

[22] Michaels A., Wu M.C., Yablonovitch E. (2020). Hierarchical Design and Optimization of Silicon Photonics. IEEE J. Sel. Top. Quantum Electron..

[23] Lee S., Hong J., Kang J., Park J., Lim J., Lee T., Jang M.S., Chung H. (2024). Inverse Design of Color Routers in CMOS Image Sensors: Toward Minimizing Interpixel Crosstalk. Nanophotonics.

[24] Park C., Lee S., Lee T., Kang J., Jeon J., Park C., Kim S., Chung H., Jang M.S. (2024). Towards Subwavelength Pixels: Nanophotonic Color Routers for Ultra-Compact High-Efficiency CMOS Image Sensors. J. Opt..

[25] Wang K., Ren X., Chang W., Lu L., Liu D., Zhang M. (2020). Inverse Design of Digital Nanophotonic Devices Using the Adjoint Method. Photonics Res..

[26] Zhao C., Cheng L., Chen H., Mao S., Wang Y., Li Q., Fu H.Y. (2023). Compact Dual-Mode Waveguide Crossing Based on Adjoint Shape Optimization. Opt. Lett..

[27] Niederberger A.C.R., Fattal D.A., Gauger N.R., Fan S., Beausoleil R.G. (2014). Sensitivity Analysis and Optimization of Sub-Wavelength Optical Gratings Using Adjoints. Opt. Express.

[28] Sapra N.V., Vercruysse D., Su L., Yang K.Y., Skarda J., Piggott A.Y., Vuckovic J. (2019). Inverse Design and Demonstration of Broadband Grating Couplers. IEEE J. Sel. Top. Quantum Electron..

[29] Miller O.D. (2012). Photonic Design: From Fundamental Solar Cell Physics to Computational Inverse Design. Ph.D. Thesis.

[30] Lalau-Keraly C.M. (2017). Optimizing Nanophotonics: From Photoreceivers to Waveguides. Ph.D. Thesis.

[31] Neustock L.T., Hansen P.C., Russell Z.E., Hesselink L. (2019). Inverse Design Tool for Ion Optical Devices Using the Adjoint Variable Method. Sci. Rep..

[32] Pan Z., Pan X. (2023). Deep Learning and Adjoint Method Accelerated Inverse Design in Photonics: A Review. Photonics.

[33] Chung Y.-S., Lee B.-J., Kim S.-C. (2009). Optimal Shape Design of Dielectric Micro Lens Using FDTD and Topology Optimization. J. Opt. Soc. Korea.

[34] Paganini A., Sargheini S., Hiptmair R., Hafner C. (2015). Shape Optimization of Microlenses. Opt. Express.

[35] Ansys Lumerical FDTD, Simulation of Photonic Components. https://www.ansys.com/products/optics/fdtd.

[36] Getting Started with LumOpt—Python API, Ansys Optics. https://optics.ansys.com/hc/en-us/articles/360050995394-Getting-Started-with-lumopt-Python-API.

[37] Kim C., Hong J., Jang J., Lee G.-Y., Kim Y., Jeong Y., Lee B. (2024). Freeform Metasurface Color Router for Deep Submicron Pixel Image Sensors. Sci. Adv..

[38] Bryce E. (1976). Bayer Color Imaging Array. U.S. Patent.

[39] Angeris G., Vučković J., Boyd S.P. (2019). Computational Bounds for Photonic Design. ACS Photonics.

[40] CMOS—Angular Response 2D, Ansys Optics Support. https://optics.ansys.com/hc/en-us/articles/360042357714-CMOS-Angular-response-2D.

[41] Akima H. (1970). A New Method of Interpolation and Smooth Curve Fitting Based on Local Procedures. J. ACM.

[42] Dyer S.A., Dyer J.S. (2001). Cubic-Spline Interpolation. 1. IEEE Instrum. Meas. Mag..

[43] Liu D.C., Nocedal J. (1989). On the Limited Memory BFGS Method for Large Scale Optimization. Math. Program..

[44] Bakr M.H. (2013). Nonlinear Optimization in Electrical Engineering with Applications in MATLAB.

[45] CMOS Image Sensor—Angular Response 3D. https://optics.ansys.com/hc/en-us/articles/360042358574-CMOS-image-sensor-Angular-response-3D.

